# The Occurrence of Oxidative Stress Induced by Silver Nanoparticles in *Chlorella vulgaris* Depends on the Surface-Stabilizing Agent

**DOI:** 10.3390/nano13131967

**Published:** 2023-06-28

**Authors:** Bruno Komazec, Petra Cvjetko, Biljana Balen, Ilse Letofsky-Papst, Daniel Mark Lyons, Petra Peharec Štefanić

**Affiliations:** 1 Department of Biology, Faculty of Science, University of Zagreb, Horvatovac 102a, 10000 Zagreb, Croatia; bruno.komazec@biol.pmf.unizg.hr (B.K.); pcvjetko@biol.pmf.unizg.hr (P.C.); bbalen@biol.pmf.unizg.hr (B.B.); 2 Institute of Electron Microscopy and Nanoanalysis (FELMI), Graz Centre for Electron Microscopy (ZFE), Austrian Cooperative Research (ACR), Graz University of Technology, Steyrergasse 17, 8010 Graz, Austria; ilse.papst@tugraz.at; 3 Center for Marine Research, Ruđer Bošković Institute, G. Paliaga 5, 52210 Rovinj, Croatia; lyons@irb.hr

**Keywords:** surface coatings, silver nanoparticles, silver ions, silver uptake, ROS content, biomolecule damage, antioxidant enzymes activity, non-enzymatic antioxidant content, ultrastructure, *Chlorella vulgaris*

## Abstract

Silver nanoparticles (AgNPs) are of great interest due to their antimicrobial properties, but their reactivity and toxicity pose a significant risk to aquatic ecosystems. In biological systems, AgNPs tend to aggregate and dissolve, so they are often stabilized by agents that affect their physicochemical properties. In this study, microalga *Chlorella vulgaris* was used as a model organism to evaluate the effects of AgNPs in aquatic habitats. Algae were exposed to AgNPs stabilized with citrate and cetyltrimethylammonium bromide (CTAB) agents and to AgNO_3_ at concentrations that allowed 75% cell survival after 72 h. To investigate algal response, silver accumulation, ROS content, damage to biomolecules (lipids, proteins, and DNA), activity of antioxidant enzymes (APX, PPX, CAT, SOD), content of non-enzymatic antioxidants (proline and GSH), and changes in ultrastructure were analyzed. The results showed that all treatments induced oxidative stress and adversely affected algal cells. AgNO_3_ resulted in the fastest death of algae compared to both AgNPs, but the extent of oxidative damage and antioxidant enzymatic defense was similar to AgNP-citrate. Furthermore, AgNP-CTAB showed the least toxic effect and caused the least oxidative damage. These results highlight the importance of surface-stabilizing agents in determining the phytotoxicity of AgNPs and the underlying mechanisms affecting aquatic organisms.

## 1. Introduction

In recent years, nanomaterials have emerged as a promising area for extensive applications in various industries with high benefits [1,2,3,4]. They play an important role in agriculture, where they are used as crop protection agents, and in medicine, where they have an antimicrobial function [5,6,7]. Among the variety of nanomaterials, silver nanoparticles (AgNPs) belong to the group that is most intensively studied and used, mainly due to their high reactivity resulting from a large surface-to-volume ratio, leading to strong biocompatibility and antibacterial and antiviral properties [8,9,10,11,12]. Due to their numerous beneficial applications, AgNPs are increasingly incorporated into many commercially available products, leading to concerns about their uncontrolled release into the aquatic environment and potentially harmful effects on the ecosystem and human health [13,14,15,16]. Due to their high reactivity, AgNPs tend to dissociate into silver ions (Ag^+^), change their shape and surface area, and agglomerate, i.e., change their size in media with high ionic strength [15].

Since their function largely depends on their physical and chemical properties, AgNPs are often stabilized with various coatings that sterically or electrostatically hinder their reactivity [17]. Many studies have shown that their stability, i.e., shape, surface area, and size, is strongly correlated with the functional properties of AgNPs and therefore plays a key role in their environmental behavior and toxicity [18,19,20,21,22,23]. Since the stability of AgNPs is of great importance, various surface coatings are used in their synthesis [17]. For this purpose, various polymers (polyvinylpyrrolidone, PVP, and polyethylene glycol, PEG), surfactants (cetyltrimethylammonium bromide, CTAB, and sodium dodecyl sulphate, SDS), polysaccharides (gum arabic, GA), and carboxylic acids (citrate) can be used [17]. However, the stabilizing agents may affect the solubility and reactivity of AgNPs [17,24], thus influencing their behavior and transformation in the exposure medium [22], which in turn affects their phytotoxic effects [25,26,27].

Studies have shown that the antimicrobial effect of AgNPs results from several mechanisms [28]. AgNPs can damage the integrity of the cell wall and lead to the inactivation of biologically important enzymes through binding of AgNPs to -SH groups of proteins [10]. Furthermore, AgNPs can improve the photocatalytic properties of other metal nanoparticles, increasing the overall antimicrobial effect [28,29]. Moreover, AgNPs can also have a genotoxic effect through direct binding to phosphoric acid residues in DNA [30,31] or through induction of the synthesis of reactive oxygen species (ROS), which can consequently destabilize other biologically important molecules [23]. Through the mentioned mechanisms, AgNPs can have significant effects on organisms such as plants and algae [32,33,34,35,36,37,38]. Since green microalgae are not only the largest oxygen producers but also the most important food producers, it is necessary to study the effects of potentially high levels of anthropogenic pollutants such as AgNPs on them [39]. Previous toxicological studies have yielded conflicting results. Treatment of the alga *Chlorella vulgaris* with uncoated AgNPs resulted in increased formation of ROS molecules and destabilization of lipids [40], while a separate study showed no changes in the level of ROS in the same alga after treatment with citrate-coated AgNPs (AgNP-citrate) [41]. Lekamge et al. [42] have shown that AgNPs coated with different ligands lead to different growth inhibition of the alga *Raphidocelis subcapitata*. A large body of research on the effect of AgNPs on microalgae has shown that they can increase or decrease the activity of certain antioxidant enzymes [33,38,41,42,43,44,45]. Zhao et al. [43] showed an increase in superoxide dismutase (SOD) activity and a decrease in peroxidase activity in *Chlamydomonas reinhardtii* after treatment with uncoated AgNPs, while increased peroxidase activity was found in *C. vulgaris* after treatment with AgNP-citrate [41].

Many studies addressing the phytotoxicity of AgNPs in different plant species have shown that ligands and coatings used to stabilize AgNPs can contribute strongly to AgNP-induced toxicity and that their effects are likely related to the intrinsic properties of the stabilizing agents [17]. However, similar studies on freshwater green algae are scarce. To our knowledge, only a few studies (six, to be exact) have compared the effects of differentially stabilized AgNPs (mostly citrate and PVP) on freshwater green algae under the same experimental conditions [42,44,46,47,48,49], and most of them investigated the effects on growth and photosynthesis [46,47,48,49], while one study investigated the activities of two antioxidant enzymes [42]. The novelty of our research is the comprehensive attempt to decipher whether and how AgNP stabilizers can cause the occurrence of oxidative stress and the activation of enzymatic and non-enzymatic antioxidants in algae, since studies in plants have shown that oxidative stress is a fundamental mechanism of AgNP-induced toxicity. Given the lack of such work in algae, it is of utmost importance to investigate the effects of AgNPs with various stabilizing agents on the formation of ROS, damage to biological macromolecules, and activation of components of the antioxidant machinery. Therefore, in this study, we investigated the possible phytotoxic effects of electrostatically stabilized AgNPs with negatively charged citrate anion (citrate) and positively charged cationic surfactant cetyltrimethylammonium bromide (CTAB) on the freshwater green algae *C. vulgaris*. Since this alga is one of the most abundant organisms in aquatic ecosystems, it is often used as a model in toxicological research. The cells of *C. vulgaris*, in the exponential growth phase of culture, were exposed to an effective concentration (EC_25_) that allows 75% of the cells to survive after 72 h of treatment with AgNP-citrate, AgNP-CTAB, or AgNO_3_ so as to analyze and compare the possible toxic effects of AgNPs with two different types of surface stabilizers and possibly differentiate the effect of Ag^+^ ions on phytotoxic mechanisms commonly associated with the adverse effects of AgNPs. Cells were analyzed in terms of silver accumulation, oxidative stress occurrence and mitigation, and ultrastructure.

## 2. Materials and Methods

### 2.1. AgNP Synthesis and Characterization

All chemicals were obtained from Sigma-Aldrich, St. Louis, MO, USA, unless otherwise stated, and were of at least analytical purity. Ultrapure water (ion-free Milli-Q water, 18.2 MΩ-cm, Merck Millipore, Billerica, MA, USA) was used for all synthesis procedures.

The syntheses of AgNP stabilized with citrate (AgNP-citrate) and cetyltrimethylammonium bromide (AgNP-CTAB) were performed as described in Peharec Štefanić et al. [26]. Briefly, the synthesis of AgNPs stabilized with citrate was performed by adding 5 mL of aqueous sodium citrate solution (1% *w*/*v*) to 120 mL of a stirred boiling aqueous solution of AgNO_3_ (99.999% purity, 0.02 g). After the color change from transparent to pale yellow, the solution was cooled to room temperature under a stream of cold water. AgNPs stabilized with CTAB were synthesized by adding 65 mL of ultrapure water containing 0.0043 g CTAB and 0.02 g AgNO_3_ to a stirred 60 mL aqueous solution containing 0.01 g ascorbic acid with a burette in a slow but constant stream. Both solutions had been cooled to 0 °C before mixing. A color change from transparent to pale orange indicated the completion of silver reduction. Both AgNP solutions were stored at 4 °C until analysis. The physical and chemical properties of the AgNP-citrate and AgNP-CTAB stock solutions were analyzed as previously described [26].

The formation of AgNPs in both solutions was confirmed by the presence of the characteristic surface plasmon resonance (SPR) peak using a UV-Vis spectrophotometer (Unicam, Cheshire, UK). The size (hydrodynamic diameter, *d*_H_) and charge (ζ-potential) of AgNPs were measured by dynamic light scattering (DLS) and electrophoretic light scattering (ELS) methods using a NanoBrook 90Plus particle size analyzer (Brookhaven Instruments, Holtsville, NY, USA) equipped with a red laser (660 nm). The intensity of the scattered light was recorded at an angle of 180° for the size measurements and at an angle of 15° for the zeta potential measurements. All measurements were performed at 25 °C. Zeta Plus software 5.71 was used for data processing. AgNPs hydrodynamic diameters are given as the average of 10 measurements (mean ± S.E., *n* = 10) and are reported as volume size distributions, while AgNP ζ-potentials are given as the average of five measurements (mean ± S.E., *n* = 5) of electrophoretic mobilities. Electrokinetic ζ-potentials are calculated from electrophoretic mobilities using the Smoluchowski equation.

The concentration of Ag^+^ ions from AgNP dissolution in ultrapure water was determined by centrifugal ultrafiltration (Millipore Amicon Ultra-4 3K, Merck Millipore, Billerica, MA, USA) of the colloid through a membrane with a molecular weight cutoff of 3 kDa. Total silver concentrations in colloidal AgNP suspensions and filtrates were determined in acidified solutions (10% HNO_3_) using an ELAN DRC-e (Perkin Elmer, Waltham, MA, USA) inductively coupled plasma mass spectrometer (ICP-MS). A calibration curve using a series of standards with known concentrations was used to calculate silver concentration. The limit of detection and the limit of quantification (LOQ) were 0.2 and 1 mg kg^−1^, respectively.

In addition, synthesized and purified AgNP-citrate and AgNP-CTAB in ultrapure water were visualized using a monochromatic TF20 (FEI Tecnai G2, FEI, Hillsboro, OR, USA) TEM with energy dispersive-X-ray (EDX) detector as in our previous study [26].

### 2.2. Chlorella vulgaris Cell Culture

A culture of the alga *C. vulgaris* was grown on liquid BBM culture medium (Bischoff and Bold, 1963 [50,51]) in pre-autoclaved sterile 200 mL Erlenmeyer flasks sealed with absorbent cotton and aluminum foil. Algae were grown in a plant growth chamber at 24 °C under long-day conditions (16 h light, 8 h dark) and a light intensity of 80 μmol m^−2^ s^−1^ with constant mixing using an orbital mixer. Algal culture was maintained by cultivating in fresh liquid BBM medium every 14 days to ensure sufficient numbers of algal cells for the experiment.

The growth of *C. vulgaris* algal culture was monitored by cell counting with an automatic cell counter (LUNA II, Logos Biosystems, Anyang, Republic of Korea) at the same time every day for a period of 30 days. For measurement with an automated cell counter, an aliquot was separated from six replicates of the algal cultures and dilutions were prepared for counting, i.e., 950 μL of distilled water was mixed with 50 μL of the algal culture. Using an automated pipette, 12 μL of the sample was added to a reusable LUNA™ slide, which was inserted into the cell counter. The final results are expressed as the concentration of cells in mL of algal suspension. The growth of the algal culture was monitored to determine the onset and duration of the exponential (log) growth phase.

### 2.3. Cell Viability Assay by Flow Cytometry

Algal culture at an initial concentration of 1 × 10^5^ cells mL^−1^ was established by inoculating the cells onto a liquid culture BBM medium in sterile Erlenmeyer flasks. The algal cells grew for 4 days in a plant growth chamber under the above-mentioned conditions with constant mixing using an orbital mixer. After 4 days of growth, the algae reached a concentration of 1 × 10^6^ cells mL^−1^ in the exponential growth phase and were exposed to a concentration range of 0.005–3.5 mg L^−1^ for AgNP-citrate and AgNO_3_ and a concentration range of 0.005–5.0 mg L^−1^ for AgNP-CTAB for 72 h according to OECD guidelines [52]. The concentrations that allowed 75% cell survival (EC_25_) after 72 h of treatment in liquid BBM medium were determined by cell viability assay using flow cytometry in combination with the fluorescent dye propidium iodide (PI) as described in [53]. Briefly, propidium iodide was added to 1 mL of cell suspension at a final concentration of 1 µg mL^−1^ and the samples were incubated for 5 min in the dark followed by flow cytometer analysis (BD FACSVerse^TM^, Piscatway, NJ, USA) using a 488 nm laser. A total of 100,000 cells per sample were analyzed. The four-parameter logistic regression (4PL) model was used [54] to determine the concentrations that lead to EC_25_. An algal culture without the addition of AgNPs served as a control sample. Three biological replicates were performed for each sample.

### 2.4. AgNP Stability in Liquid BBM Medium

The stability of AgNP-citrate, AgNP-CTAB, and AgNO_3_ at concentrations leading to EC_25_ in a liquid BBM culture medium was determined by UV-Vis spectroscopy, as described in Peharec Štefanić et al. [55]. Changes in the position and the intensity of the SPR peak of prepared suspensions kept under the same conditions as the algal cell cultures were observed after 1, 2, 3, 4, 5, 24, 48, and 72 h. To better understand the initial transformations of AgNPs in the culture medium, the changes in *d*_H_ and ζ-potential were observed by the DLS method on the NanoBrook 90Plus particle size analyzer at the same time intervals as for UV-Vis. Results are presented as volume size distributions and represent mean value ± S.E. of 10 measurements. The ζ-potentials are given as mean value ± S.E. of 5 measurements.

### 2.5. Silver Content Measurements

The measurement of silver content in the treated algal cells was performed as previously reported [25]. Briefly, the algal cell culture was centrifuged at 3500× *g* for 3 min. The obtained pellet was resuspended in 10 mL of buffer consisting of 2 mM Na_2_HPO_4_·12H_2_O, 4 mM NaH_2_PO_4_·H_2_O, 9 mM NaCl, and 1 mM KCl at pH 7.0, and 5 g of 20–50 mesh Amberlite HPR1100 ion exchange resin (Sigma-Aldrich, St. Louis, MO, USA) was added to remove possible impurities on the cell surface that could interfere with the measurement of internalized AgNPs. After stirring at 4 °C for 2 h, the homogenate was centrifuged at 4500× *g* for 30 min. The obtained pellet was washed thoroughly with 1× PBS buffer (10 mM Na_2_HPO_4_, 1.7 mM KH_2_PO_4_, 2.7 mM KCl, 130 mM NaCl) and centrifuged again. The pellet was then freeze-dried for 24 h. The cells in the pellet were later digested in a microwave oven (ETHOS SEL Milestone, Shelton, CT, USA) according to the method EPA 3051a—first in 10 mL of concentrated nitric acid (HNO_3_) at 130 °C for 10 min, then at 180 °C for another 15 min. The second step was digestion in 1 mL of hydrogen peroxide (H_2_O_2_) at 85 °C for 5 min and then at 130 °C for 4 min. Samples were cooled and then diluted with 1% (*v*/*v*) HNO_3_ to a final volume of 50 mL. The ELAN DRC-e ICP-MS instrument was used to determine the total silver content. A calibration curve using a series of standards with known concentrations was used to calculate the silver concentration. The limit of detection and the limit of quantification (LOQ) were 0.05 and 0.1 mg kg^−1^, respectively. The spike recoveries were 95.6%, 95.2%, and 96.5%. Silver content was expressed in micrograms of silver per 10^6^ cells.

### 2.6. Protein Extraction

Total soluble proteins were extracted from a suspension of algal cells grown on BBM culture medium after being treated for 72 h with concentrations of AgNP-citrate and AgNP-CTAB and AgNO_3_ that allowed 75% cell survival, and from control groups of cells. To prepare the protein extracts, 200 mL of cell suspension was centrifuged at 3500× *g* and 20 °C for 5 min. After centrifugation, the supernatant was discarded, and the pellet was washed three times with ultrapure water. After each wash, the cells were centrifuged at 3500× *g* and 20 °C for 3 min. After the washing cycle, cells were homogenized using a Retsch homogenizer (MM200, Retsch, Haan, Germany) at 30 Hz for 3 min at 4 °C, adding glass beads (425–600 µm) in 500 µL of 50 M potassium phosphate buffer at pH 7.0. The homogenates obtained were centrifuged at 20,000× *g* and 4 °C for 15 min, and the supernatant was transferred to clean tubes and centrifuged again for 45 min under the same conditions. Protein concentration was determined according to the Bradford assay [56], using bovine serum albumin (BSA) as a standard. The obtained extracts were used for the quantification of protein carbonyl and the study of enzymatic activities.

### 2.7. Reactive Oxygen Species Determination

Two fluorescent probes were used to determine the total ROS content: dihydroethidium (DHE) according to the modified protocol described in Cvjetko et al. [25] and 2,7-dichlorodihydrofluorescein diacetate (H_2_DCFDA) following the modified protocol described in Ng et al. [57]. Briefly, 80 μL of 20 μM DHE or H_2_DCFDA was added to 100 μL of cell suspension in a 96-well microtiter plate. The cell suspensions were incubated in the dark for 30 min, and fluorescence was then measured at 520 nm excitation and 600 nm emission or at 504 nm excitation and 550 nm emission for DHE or H_2_DCFDA using a GloMax microplate reader (Promega, Madison, WI, USA). The results were then normalized to the number of cells in the cell suspension and expressed as a percentage of the ROS content in the control cells.

H_2_O_2_ content was measured according to a modified protocol described in Mátai et al. [58]. Briefly, 200 mL of cell suspension was centrifuged at 3500× *g* at 20 °C for 5 min, after which the supernatant was discarded and the pellet was washed three times with ultrapure water. After each wash, the cells were centrifuged again under the same conditions. The resulting cell pellet was resuspended in 70% ethanol and the cells were homogenized using a Retsch homogenizer (MM200, Retsch, Haan, Germany) at 30 Hz for 3 min at 4 °C, adding glass beads (425–600 µm). The homogenates were then centrifuged at 15,000× *g* for 10 min at 4 °C, and the supernatant was transferred to clean vials and stored on ice. The reaction mixture consisted of 1000 μL 124 μM xylenol orange sodium salt, 99 mM sorbitol, and 0.248 mM ammonium iron (II) sulphate, to which 100 μL of the prepared samples was added. The reaction mixture was then vortexed and incubated at room temperature for 15 min, followed by absorbance measurement at 560 nm. The total amount of H_2_O_2_ was determined using known standards, and the results were expressed as μmol of H_2_O_2_ per 10^6^ cells.

### 2.8. Malondialdehyde and Protein Carbonyl Content

The level of lipid peroxidation in the algae was determined by measuring malondialdehyde (MDA) content using a modified protocol as described in Heath and Packer [59]. First, 200 mL of the cell suspension was centrifuged at 3500× *g* at 20 °C for 5 min, after which the pellet was washed three times with ultrapure water and centrifuged again under the same conditions. The cell pellet was homogenized three times with a MM200 Retsch homogenizer at 30 Hz for 3 min, adding glass beads (425–600 µm) in 700 µL of 0.5% (*w*/*v*) 2-thiobarbituric acid prepared in 20% (*w*/*v*) trichloroacetic acid (TCA). The obtained extracts were incubated at 95 °C for 30 min. Subsequently, the samples were centrifuged at 14,000× *g* for 30 min at 4 °C and their absorbance was measured at 532 nm. In addition, the absorbance at 600 nm was measured and then subtracted from the value measured at 532 nm to correct for nonspecific turbidity. For the final calculation of MDA content, the values obtained were normalized to the same number of cells between samples and the results were expressed as a percentage of control cells.

Protein carbonyls were quantified by the modified method of Levine et al. [60], which uses a reaction with 2,4-dinitrophenylhydrazine (DNPH). Briefly, 180 μL of 100 mM potassium phosphate buffer (pH 7.0) was added to 20 μL of protein extract (Section 2.6), and the resulting solution was mixed with 300 μL of 10 mM DNPH in 2 M HCl or with 300 μL of 2 M HCl. In such treatment, each sample has its own reference, and all samples are incubated for 1 h at room temperature in the dark. After incubation, proteins were precipitated by adding 500 μL of cold 10% (*w*/*v*) TCA, samples were cooled to −20 °C, and centrifuged at 20,000× *g* and 4 °C for 10 min. The supernatant was discarded, and the sediments were washed three times with 500 µL ethanol/ethyl acetate mixture (1/1: *v*/*v*). Proteins were then dissolved in 6 M urea in 20 mM potassium phosphate buffer (pH 2.4) in an ultrasonic bath. The absorbance was measured at 370 nm, while the absorbance of each sample was measured at 280 nm to evaluate protein recovery. The final result of protein carbonyl content was calculated using the molar absorption coefficient for aliphatic hydrazone of 22 mM^−1^ cm^−1^ and expressed in micromoles per 10^6^ cells.

### 2.9. Comet Assay

The Comet assay was performed according to the protocol published in Gichner et al. [61] with modifications published in Cvjetko et al. [25]. Briefly, 100 μL of cell suspension was mixed with 100 μL of 1% (*w*/*v*) low melting point (LMP) agarose. Samples were then denatured in lysis buffer (2.5 M NaCl, 100 mM ethylenediaminetetraacetic acid (EDTA), 10 mM Tris-HCl, 10% DMSO, 1% Triton X-100) for 60 min at 4 °C, followed by electrophoresis for 20 min (0.8 V cm^−1^ and 300 mA) in a buffer of 1 mM Na_2_EDTA and 300 mM NaOH (pH 13). After electrophoresis, slides were neutralized with 0.4 M Tris-HCl, air-dried, and then stained with a nucleic acid stain (GelStar™ Nucleic Acid Gel Stain, Lonza, Basel, Switzerland). DNA damage was measured by analyzing the percentage of DNA tail using the OpenComet tool of ImageJ software 1.53t [62].

### 2.10. Activity Assays of Antioxidant Enzymes

Enzyme kinetics analysis was performed at room temperature using a UV/Vis spectrophotometer (ATI UNICAM UV4, Cambridge, UK) for spectrophotometric analysis.

SOD (E.C. 1.15.1.1) activity was determined according to the protocol used by Beauchamp and Fridovich [63]. Different volumes of protein extracts were added to the reaction mixture (13 mM methionine, 0.1 M EDTA, 75 µM nitroblue tetrazolium (NBT), and 2 mM riboflavin) containing 50 mM potassium phosphate buffer (pH 7.8). Samples were then incubated at room temperature for 8 min in a 15 W light box, and formazan produced by NBT photoreduction was measured at 560 nm. SOD activity is defined as the amount of enzyme that causes 50% inhibition of the NBT reduction rate, which corresponds to one unit of SOD activity, and the results are expressed as U mg^−1^ proteins.

Pyrogallol peroxidase (PPX, EC 1.11.1.7) activity was determined according to the protocol used by Nakano et al. [64]. Briefly, protein extract was added to the reaction mixture (50 mM potassium phosphate buffer, pH 7.0, 20 mM pyrogallol, and 5 mM H_2_O_2_) at a final concentration of 2% (*v*/*v*). The presence of the PPX enzyme in the sample resulted in an increase in absorbance at 430 nm due to pyrogallol oxidation (ε = 2.6 mM^−1^ cm^−1^). Specific PPX activity was expressed as µmol purpurogallin min^−1^ mg^−1^ proteins.

Ascorbate peroxidase (APX, EC 1.11.1.11) activity was determined by a modified method described in Nakano and Asada [64]. Briefly, protein extract was added to the reaction mixture (50 mM potassium phosphate buffer, pH 7.0, 0.5 mM ascorbate, and 10 mM H_2_O_2_) at a final concentration of 18% (*v*/*v*) and the decrease in absorbance at 290 nm was measured (ε = 2.8 mM^−1^ cm^−1^). Specific activity of APX was expressed as µmol oxidized ascorbate min^−1^ mg^−1^ proteins.

Catalase activity (CAT) (EC 1.11.1.6) was determined according to a modified protocol described in Aebi [65]. Briefly, protein extract was added to the reaction mixture (50 mM potassium phosphate buffer, pH 7.0, 20 mM H_2_O_2_) at a final concentration of 5% (*v*/*v*) and then the decrease in absorbance at 240 nm was measured (ε = 36 mM^−1^ cm^−1^), and the results were expressed as µmol decomposed H_2_O_2_ min^−1^ mg^−1^ proteins.

### 2.11. Assays of Non-Enzymatic Antioxidants

For analysis of proline and glutathione content, 200 mL of the cell suspension was centrifuged at 3500× *g* and 20 °C for 5 min, after which the pellets were washed with ultrapure water and finally resuspended in 3% 5-sulfosalicylic acid (SA). The samples were homogenized as described in Section 2.6. Extracts were centrifuged at 10,000× *g* and 4 °C for 15 min, and the supernatants were transferred to clear vials and stored on ice until analysis.

Proline content was determined according to the protocol described in Bates and Waldren [66]. Briefly, 500 µL of the extract was mixed with 500 µL of glacial acetic acid and 500 µL of acidic ninhydrin, and the samples were incubated at 95 °C for 1 h. After incubation, the samples were mixed with 1.2 mL of toluene and the absorbance of the toluene layer was measured at 520 nm. The final concentration of proline is expressed as µmol proline per 10^6^ cells, which was determined using standards with known concentrations.

Glutathione level was determined according to the method of Salbitani et al. [67]. First, the amount of reduced glutathione was measured by mixing 100 µL of the extract with 750 µL of the reaction mixture (0.1 M potassium phosphate buffer pH 7.0, 1 mM EDTA, 1.5 mg mL^−1^ 5,5-dithio-bis-(2-nitrobenzoic acid)) and incubating the samples at room temperature for 20 min before measuring the absorbance at 412 nm. Then, 50 µL of nicotinamide adenine dinucleotide phosphate (NADPH) (0.32 mg mL^−1^) and 15 µL of glutathione reductase (1 U) were added to each sample and the samples were incubated for 5 min before total glutathione was determined by measuring the absorbance at 412 nm. Glutathione concentration is expressed as moles of glutathione per 10^6^ cells, using standards of known concentrations. The amount of oxidized glutathione was calculated as the difference between total and reduced glutathione, and the results were expressed as the ratio of reduced (GSH) and oxidized (GSSG) glutathione.

### 2.12. Ultrastructural Analyses

Ultrastructural changes were observed with a transmission electron microscope (TEM). Cell cultures were first centrifuged at 3500× *g* for 3 min at room temperature. The resulting pellet was fixed with 500 µL 1% (*w*/*v*) glutaraldehyde in 50 mM cacodylate buffer (pH 7.2), and 50 µL was aliquoted into clean tubes. Cooled (but not solidified) 1.5% (*w*/*v*) agarose was added to the 50 µL of fixed algal cells and the mixture was resuspended. After solidification, the agarose pieces with the embedded cells were fixed at 4 °C for another 1.5 h. Samples were then washed twice with cold 50 mM cacodylate buffer (pH 7.2) and postfixed with 1% (*w/v*) osmium tetroxide in the same buffer for 1.5 h at 4 °C. After postfixation, samples were washed twice with ultrapure water for 10 min at 4 °C. Samples were then dehydrated in a graded series of ethanol, and finally, the agarose pieces with the cells, were embedded in Spurr’s resin. Commercially available UranyLess (Em-Grade.com, Mauressac, France) and 3% (*w/v*) Reynolds lead citrate (Em-Grade.com, Mauressac, France) were used only for staining the ultrathin sections of the control cells. Ultrathin sections of algal cells treated with AgNP-citrate, AgNP-CTAB, and AgNO_3_ were not positively contrasted. Ultrathin sections were examined using a monochromatic TF20 (FEI Tecnai G2) TEM.

### 2.13. Statistical Analysis

Statistical analysis was performed by one-way ANOVA followed by Newman–Keuls post hoc test using the STATISTICA 14.0.0.15 software package (TIBCO Software, Inc., Palo Alto, CA, USA). Differences between means were considered statistically significant at *p* ≤ 0.05.

## 3. Results

### 3.1. AgNP Characterization

Stock solutions of laboratory synthesized AgNP-citrate and AgNP-CTAB in ultrapure water were characterized by UV-Vis spectroscopy, TEM, DLS, and ELS, and the results obtained are listed in the Supplementary Materials. UV-Vis spectra showed SPR peaks at 418 nm for AgNP-citrate (Figure S1A) and 449 nm for AgNP-CTAB (Figure S1E), confirming the synthesis of AgNPs with 47 nm and 72 nm nominal diameters, respectively. DLS measurements confirmed the success of the synthesis, and the average *d*_H_ of the obtained nanoparticles were determined to be 41.4 ± 0.9 nm for AgNP-citrate and 82.8 ± 1.1 nm for AgNP-CTAB (Table S1). TEM microphotographs showed that both AgNP-citrate (Figure S1B) and AgNP-CTAB (Figure S1F) were generally spherical in shape with a small proportion of rod-shaped particles. In addition, it was observed from the TEM images that the particles stabilized with citrate had a diameter of 40–60 nm, while the particles stabilized with CTAB were slightly larger and mostly had a size of 50–90 nm. The silver elemental map (Figure S1C,G) combined with energy dispersive X-ray microanalysis (Figure S1D,H) confirmed that all detected particles contained silver. The value of ζ-potential of AgNP-citrate stock solution was −40.50 ± 3.21 mV, while that of AgNP-CTAB stock solution was 51.34 ± 2.05 mV. The concentration of AgNP-citrate and AgNP-CTAB stock solutions determined by ICP-MS was 112.2 and 94.6 mg L^−1^, respectively, while the amount of ionic Ag in the synthesized dispersions was 0.5% in both AgNP stock solutions (Table S1).

### 3.2. Cell Viability Assay by Flow Cytometry

**Figure 1:**
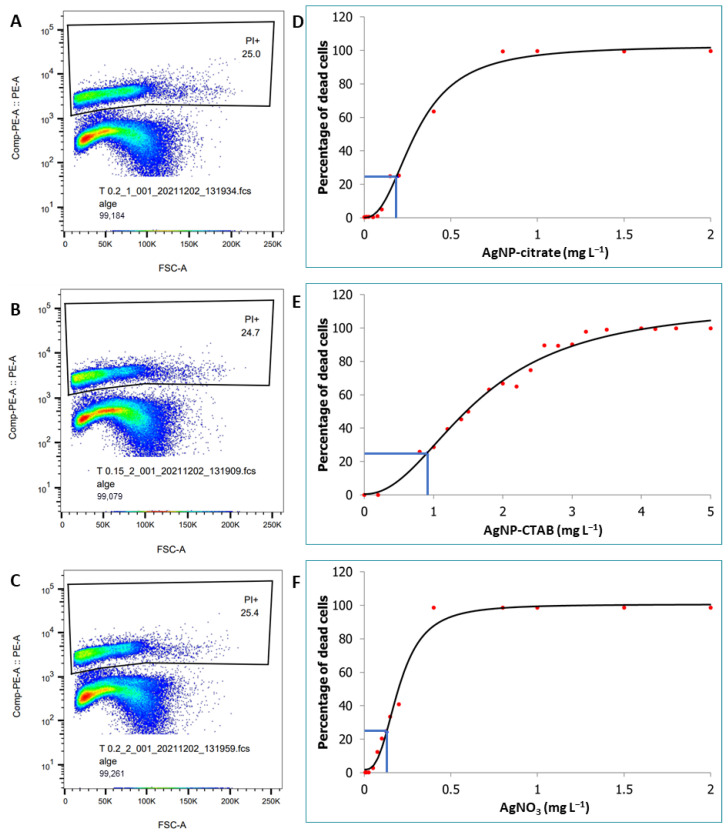
Cell viability after 72 h treatment with increasing concentrations of differently stabilized AgNPs or with AgNO_3_ in liquid BBM culture medium was determined by cell survival analysis using flow cytometry in combination with the fluorescent dye propidium iodide (PI). Flow cytometry images were obtained for each concentration. The flow cytometry images presented here show nearly 25% dead cells (**A**–**C**). The four-parameter logistic regression (4PL) model was used to determine the concentrations leading to EC_25_ (**D**–**F**). The concentrations determined by this model were used for all experiments. Values represent the means of two different experiments, each with three replicates (*n* = 6). Blue line represents the EC_25_.

The curves of cell viability after 72 h of treatment with increasing concentrations of differently stabilized AgNPs or with AgNO_3_ in liquid BBM culture medium, as determined by cell survival analysis using flow cytometry in combination with the fluorescent dye PI, are shown in Figure 1. The 4PL model was used to determine the concentrations at which 75% of cells survive (EC_25_). According to the results, differently stabilized AgNPs had different effects on algal cell death. Treatment with AgNP-citrate (Figure 1A,D) resulted in faster cell death than treatment with AgNP-CTAB (Figure 1B,E), which was found to be the least toxic of all treatments applied. The calculated EC_25_ values were 0.188 mg L^−1^ and 0.895 mg L^−1^, respectively. The treatment with AgNO_3_ (Figure 1C,F) resulted in the fastest algal death compared to both types of nanoparticles, with a calculated EC_25_ value of 0.130 mg L^−1^.

### 3.3. AgNP Stability in Liquid BBM Medium

The stability of AgNP-citrate, AgNP-CTAB, and AgNO_3_ in liquid BBM culture medium was analyzed by UV/Vis spectroscopy; the results are shown in Figure S2. Although the AgNPs studied had different stabilizing agents, they tended to agglomerate rapidly after contact with the culture medium.

The results show that the addition of AgNP-citrate to the BBM medium resulted in a shift of the SPR peak to higher wavelengths compared to the peak of the stock solution (from 411 nm to 467 nm) after only 1 h, demonstrating the initial agglomeration of the nanoparticles (Figure S2A). Moreover, after 3 h, a slight shift in the SPR peak to shorter wavelengths (from 469 nm to 435 nm) was observed, indicating a global decrease in the diameter of the nanoparticles. After 3 h and until the end of 72 h, the position of the SRP peaks remained relatively constant. Moreover, the intensity of the SRP peak of AgNP-citrate in the medium increased up to 5 h, then decreased up to 24 h and stabilized until the end of 72 h. This result indicates the initial reduction of Ag^+^ ions to elemental silver (Ag^0^) after 5 h and their subsequent dissociation to Ag^+^ ions.

Similar to AgNP-citrate, the intensity of the SRP peak of AgNP-CTAB shifted dramatically to higher wavelengths (from 446 nm to 473 nm) after the addition of nanoparticles to the medium, indicating the initial agglomeration of AgNP-CTAB (Figure S2B). After 3 h, a slight shift of the SRP peaks toward shorter wavelengths (from 467 nm to 426 nm) was observed, describing the decrease in AgNP-CTAB diameter. The position of the SRP peak did not change significantly until the end of 72 h. Analysis of the intensity of the SPR peaks showed that AgNP-CTAB in the BBM medium was stable until the third hour, with a slight decrease in the intensity of the SPR peak, but a sharp increase thereafter. After that, the intensity of the SPR peak slightly decreases until 48 h and slightly increases again until 72 h. These changes in the intensity of the SPR peaks indicate the reduction of Ag^+^ ions and the formation of AgNPs by the third hour, after which a relative stabilization occurs until the end of the analysis.

The addition of AgNO_3_ as a source of Ag^+^ ions to the BBM medium resulted in the formation of AgNPs within the first hour, as shown by the appearance of the characteristic SPR peak at 485 nm (Figure S2C). At the end of the second hour, the position of the SPR peak shifted toward shorter wavelengths (457 nm), indicating a global decrease in the diameter of the newly formed AgNPs, and there were no further significant shifts in the SPR peak until the end of the analysis. Moreover, the intensity of the SPR peak caused by the interaction of Ag^+^ ions from AgNO_3_ and ions for the elements in the BBM medium gradually increased until the fourth hour, then decreased until the 48 h, and finally stabilized until the 72 h. This result indicates that Ag^+^ ions react strongly with other ions in an ion-rich culture medium, which inevitably leads to the synthesis and dissociation of AgNPs.

DLS measurements further confirmed the agglomeration by showing a shift in the volume size distribution toward larger *d*_H_ values after addition of AgNP to the medium compared to stock solutions (Table S2). More specifically, DLS measurements for AgNP-citrate and AgNP-CTAB showed that the nanoparticles agglomerated immediately in the medium, with diameters of 109 nm and 106 nm, respectively. AgNP-citrate remained stable up to 24 h, then the agglomerate diameter gradually decreased to 61 nm after 72 h. The AgNP-CTAB agglomerates that were present in the medium remained similar in size until the end of 72 h. The DLS results for AgNO_3_ agree well with the UV/VIS spectroscopy results and show the de novo synthesis of AgNPs, whose size decreased from an initial 174 nm to 94 nm after 24 h and further slightly decreased to 84 nm at the end of 72 h, which is also visible in the UV/VIS spectrum.

Zeta potential analysis showed that AgNP-citrate adds its negative charge to the total charge of the particles in the medium, and the charge remains negative until the end of 48 h, after which it becomes positive. On the other hand, the positive charge of AgNP-CTAB is lost in the total charge of the particles in the medium at the very beginning and remains negative until the end of the measurement. Newly formed AgNPs, resulting from the interaction between the salt present in the BBM medium and AgNO_3_, resulted in a net negative charge on the surface of the particles in the medium until the seventh hour, after which the total charge of the particles in the medium was zero.

### 3.4. Silver Content Measurements

Analysis of Ag content in *C. vulgaris* cells showed that silver was internalized into algal cells after all treatments with AgNPs and AgNO_3_ (Table 1). The most significant silver uptake was observed upon exposure to AgNP-CTAB (26.81 µg/10^6^ cells), while the lowest accumulation was measured in algal cells treated with AgNO_3_ (9.96 µg/10^6^); the silver uptake of 15.62 µg/10^6^, obtained upon treatment with AgNP-citrate, was significantly higher compared to the values obtained with AgNO_3_, but at the same time significantly lower compared to AgNP-CTAB. The concentration of accumulated silver in the cells after the different treatments correlated well with the EC_25_ value obtained for each treatment.

### 3.5. Induction of ROS Formation

**Figure 2:**
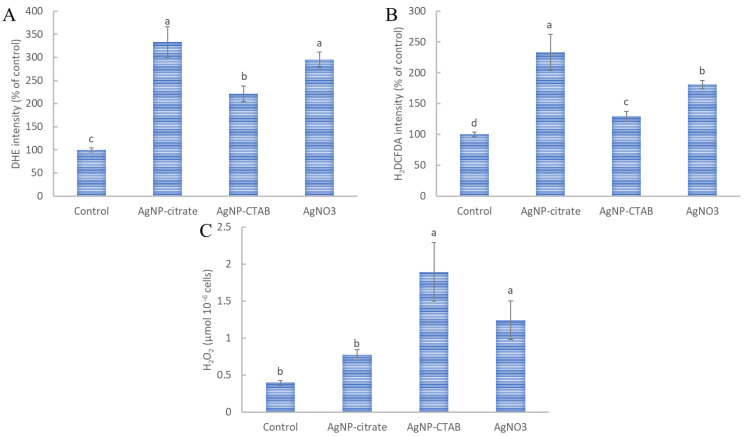
Total reactive oxygen species (ROS) content was determined in situ with (**A**) dihydroethidium (DHE) and (**B**) 2,7-dichlorodihydrofluorescein diacetate (H_2_DCFDA) in *C. vulgaris* cells 72 h after exposure to 0.188 mg L^−1^ AgNP-citrate, 0.895 mg L^−1^ AgNP-CTAB, and 0.130 mg L^−1^ AgNO_3_. Hydrogen peroxide (H_2_O_2_) content (**C**) was measured in *C. vulgaris* cell extracts 72 h after exposure to the same concentrations. Values represent the mean ± SE of two different experiments, each with 6 replicates (*n* = 12). Treatments significantly different at *p* ≤ 0.05 (one-way ANOVA followed by a Newman–Keuls post hoc test) are indicated with different letters.

All three treatment types significantly increased the production of total ROS (Figure 2A,B), whereas AgNP-CTAB and AgNO_3_ significantly increased H_2_O_2_ production compared with the control (Figure 2C). Analysis of the total content of ROS in situ using two fluorescent probes (DHE and H_2_DCFDA) showed a similar trend, with AgNP-citrate treatment leading to the highest values, although it was not significantly different for DHE compared with the results obtained with AgNO_3_; for both parameters, exposure to AgNP-CTAB led to significantly lower values (Figure 2A,B). On the other hand, AgNP-CTAB caused a prominent increase in H_2_O_2_ production compared to AgNO_3_ and especially to AgNP-citrate, whose values were not significantly different from those of the control (Figure 2C).

### 3.6. Oxidative Effect of AgNP on Lipids, Proteins and DNA

**Figure 3:**
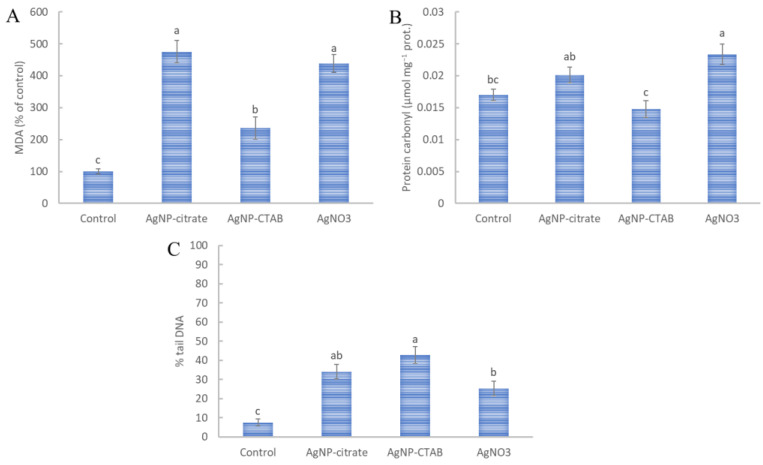
The content of (**A**) malondialdehyde (MDA) and (**B**) protein carbonyls and percentage of tail DNA (**C**) were measured in *C. vulgaris* cells 72 h after exposure to 0.188 mg L^−1^ AgNP-citrate, 0.895 mg L^−1^ AgNP-CTAB, and 0.130 mg L^−1^ AgNO_3_. Values represent the mean ± SE of two different experiments, each with six replicates (*n* = 12). Treatments significantly different at *p* ≤ 0.05 (one-way ANOVA followed by a Newman–Keuls post hoc test) are indicated with different letters.

Similar to the formation of total ROS, all applied treatments caused increased destabilization of lipids, with AgNP-citrate and AgNO_3_ causing the highest lipid peroxidation, 478% and 435% more, respectively, than in control cells (Figure 3A). On the other hand, AgNP-CTAB treatment increased MDA value by 237% compared with the control, which was significantly lower compared to treatments with AgNP-citrate and AgNO_3_.

Of all the treatments applied, only AgNO_3_ significantly increased the amount of protein carbonyls compared with the control (Figure 3B). Exposure to AgNP-citrate and AgNP-CTAB resulted in a slight increase and decrease, respectively, although these values were not statistically significant compared with the control (Figure 3B). On the other hand, the results obtained for AgNP-CTAB were significantly lower compared to AgNP-citrate and AgNO_3_.

Genotoxicity analysis revealed that all treatments increased DNA damage in *C. vulgaris* cells (Figure 3C). AgNP-CTAB showed the significantly highest genotoxic effect compared to the control, with a percentage of DNA damage of 42.77%. AgNP-citrate resulted in a DNA tail percentage of 34.10%, while AgNO_3_ showed the lowest genotoxicity compared to the other treatments, with a DNA tail percentage of 25.28% (Figure 3C).

### 3.7. Antioxidant Enzymes Activity

**Figure 4:**
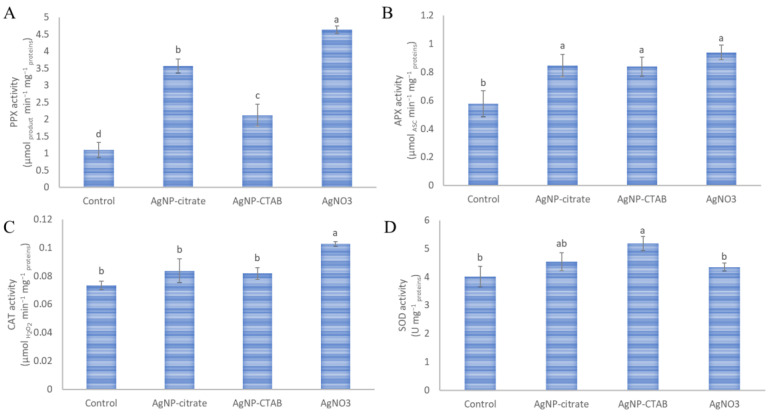
The activities of the antioxidant enzymes (**A**) pyrogallol peroxidase (PPX), (**B**) ascorbate peroxidase (APX), (**C**) catalase (CAT), and (**D**) superoxide dismutase (SOD) were measured in *C. vulgaris* cells 72 h after exposure to 0.188 mg L^−1^ AgNP-citrate, 0.895 mg L^−1^ AgNP-CTAB, and 0.130 mg L^−1^ AgNO_3_. Values represent the mean ± SE of two different experiments, each with six replicates (*n* = 12). Treatments significantly different at *p* ≤ 0.05 (one-way ANOVA followed by a Newman–Keuls post hoc test) are indicated with different letters.

All applied treatments significantly increased the activities of peroxidases compared with the control (Figure 4A,B). AgNO_3_ resulted in the significantly highest and AgNP-CTAB in the significantly lowest increase in PPX activity among the applied treatments (Figure 4A). The activity of APX was equally increased in all treatments compared with the control (Figure 4B). On the other hand, the activity of CAT was only slightly increased in both AgNP treatments, while exposure to AgNO_3_ resulted in a significant increase compared to the control (Figure 4C). In contrast, activity of SOD was increased only in the AgNP treatments, although only AgNP-CTAB resulted in a significant increase compared to the control (Figure 4D).

### 3.8. Non-Enzymatic Antioxidants

**Figure 5:**
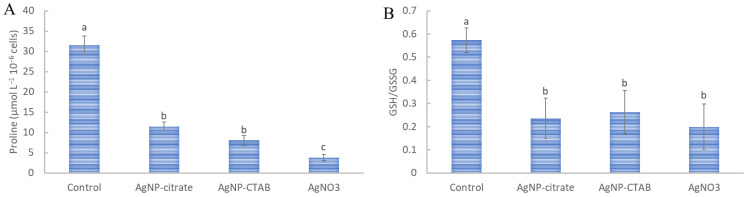
The content of (**A**) non-enzymatic antioxidant proline and (**B**) the ratio of reduced and oxidized glutathione (GSH/GSSG) were measured in *C. vulgaris* cells 72 h after exposure to 0.188 mg L^−1^ AgNP-citrate, 0.895 mg L^−1^ AgNP-CTAB, and 0.130 mg L^−1^ AgNO_3_. Values represent the mean ± SE of two different experiments, each with six replicates (*n* = 12). Treatments significantly different at *p* ≤ 0.05 (one-way ANOVA followed by a Newman–Keuls post hoc test) are indicated with different letters.

Non-enzymatic antioxidants showed decreased levels after all treatments compared to the control. All treatments caused a significant decrease in proline content, which was similar for AgNP treatments and very pronounced, and significantly the lowest for AgNO_3_ (Figure 5A). On the other hand, all treatments caused a statistically equal decrease in GSH/GSSG ratio, i.e., a significant increase in the oxidized form of glutathione compared with the control (Figure 5B).

### 3.9. Ultrastructure Analysis

**Figure 6:**
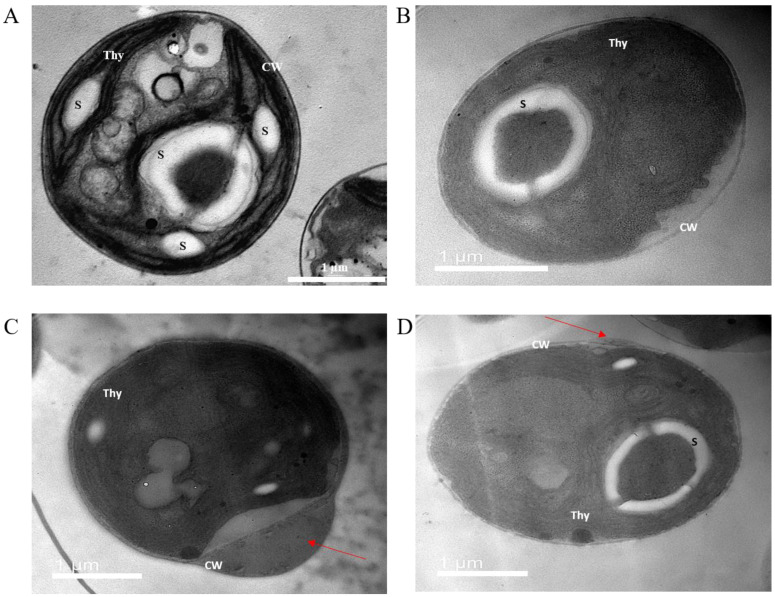
Ultrastructure of (**A**) control cells of *C. vulgaris* and cells after treatment with (**B**) AgNP-citrate, (**C**) AgNP-CTAB, or (**D**) AgNO_3_ imaged by transmission electron microscopy. Thy—thylakoids; S—starch; CW—cell wall; red arrows indicate changes in the cell wall. Bar = 1 µm.

Transmission electron microscopy showed that all treatments resulted in changes in the ultrastructure of *C. vulgaris* cells (Figure 6), which manifested as plasmolysis and instability of the cell wall. Plasmolysis was most pronounced after AgNP-citrate treatment (Figure 6B), although some degree of plasmolysis was also observed after AgNP-CTAB exposure (Figure 6C). Cell wall instability was most pronounced after treatment with AgNP-CTAB (Figure 6C), although some degree of cell wall destabilization was also observed after exposure to AgNO_3_ (Figure 6D).

## 4. Discussion

In this study, we investigated the potential phytotoxic effects of AgNPs stabilized with a negatively charged citrate anion (citrate) and a positively charged cationic surfactant (CTAB) on the freshwater green alga *C. vulgaris*. To distinguish between the effects of the nanoparticulate and ionic forms of silver, treatments with AgNO_3_, a source of Ag^+^ ions, were also included. To investigate the most realistic effects of AgNPs on algae, we used environmentally relevant concentrations of silver that allowed 75% cell survival after 72 h in liquid BBM culture medium. Indeed, studies have shown that silver concentrations in Malaysian surface waters are as high as 0.505 mg L^−1^ [68] or as high as 10.16 mg L^−1^ [69], depending on the proximity to different factories, while previous predictions based on mathematical models estimate the upper limit of global silver concentration in environmental waters to be as high as 17 µg L^−1^ [70]. Considering that more than 130 tons of silver enter European aquatic ecosystems every year, 15% of which is in the form of AgNPs [71], and that silver production is increasing each year [71,72], the concentrations we use correspond to those that can realistically occur in the environment.

The influence of the physical and chemical properties of AgNPs on their stability and subsequent biological interactions has been confirmed in several studies, especially highlighting the size of AgNPs as a crucial factor for their phytotoxicity [15,49,73,74]. However, the number of studies investigating the effects of differently coated AgNPs on living organisms is increasing [11,17,25,27,33,75]. Stabilizing agents can alter AgNP surfaces and thus affect their behavior and transformation in the exposure medium [22]. In addition, the surface stabilizing agents also determine the size and shape of the particles and affect their solubility, reactivity, and overall stability [24,32]. Studies on the phytotoxic effects of differentially coated AgNPs have mainly used aquatic and terrestrial plants as model organisms [11,17,25,27,75,76,77], while toxic effects on the growth and physiology of freshwater algae are generally much less documented [33,42,44,47,48,49,78]. To date, ecotoxicological studies of AgNPs on algae have mainly focused on uncoated AgNPs or AgNPs coated with a single stabilizer, as described in Biba et al. [17]. Few studies on algae have compared the effects of various AgNP coatings in the same experiment [42,44,47,48,79,80] and mostly found that differently coated AgNPs elicit different responses on algal growth. In our study, differently stabilized AgNPs showed different effects on algal cell death, as the EC_25_ value after 72 h for AgNP-CTAB was about four times higher than that for AgNP-citrate, suggesting that AgNPs stabilized with CTAB are less toxic than AgNP-citrate to *C. vulgaris* growing in BBM culture medium. Different effects of positively and negatively charged AgNP stabilizers on *C. vulgaris* growth were also reported by Zhang et al. [79], where negatively charged citrate-coated AgNPs resulted in a higher EC_50_ value compared to positively charged polyethylene-coated ones in BG-11 culture medium, suggesting that AgNPs with positively charged surface stabilizers were more toxic under their experimental conditions. In addition, Lekamge et al. [42] reported that AgNPs coated with negatively charged curcumin and epigallocatechin gallate inhibited *Raphidocelis subcapitata* growth at a lower concentration than those coated with neutral L-tyrosine. Taken together, these results suggest that the use of surface-stabilizing agents can significantly affect the toxicity of AgNPs to algae in terms of growth inhibition and suggest that there is a great need for comparative studies on the toxicity of differently stabilized AgNPs to the same organism under the same experimental conditions.

The different effects of AgNP-citrate and AgNP-CTAB on the growth of *C. vulgaris* obtained in this study might be related to the differences in their physicochemical properties, which are known to influence their behavior in the environment depending on the composition of the exposure medium [23,24,81]. Although tested AgNPs were stabilized with different agents, they both tended to agglomerate rapidly after exposure to the culture medium, and their surface charge also changed. However, these changes were much more pronounced in the AgNP-CTAB particles, which exhibited a stronger agglomeration effect compared to AgNP-citrate (especially after 24, 48, and 72 h), suggesting that a smaller amount of AgNP-CTAB was present in the size that can cause toxic effects on algae. This conclusion is consistent with the results obtained for AgNO_3_, which indicate that Ag^+^ ions released from AgNO_3_ reacted with ions from the ion-rich BBM culture medium, resulting in the synthesis of newly formed AgNPs, whose behavior in terms of changes in *d*_H_ and surface charge was similar to that of AgNP-citrate. Moreover, treatment with AgNO_3_ resulted in the fastest death of algal cells compared to both AgNP types, with a calculated EC_25_ value of 0.130 mg L^−1^, which is consistent with other studies [42,80,82] and suggests that the effects of AgNO_3_ are due to both the newly formed AgNPs and Ag^+^ ions.

The AgNP citrate and AgNP-CTAB used in this study had different initial diameters (47 and 72 nm, respectively), the size of which could also affect their phytotoxicity. Indeed, algal cells have a porous cell wall structure [83,84] that facilitates adsorption and allows internalization of AgNPs up to 20 nm in diameter under normal conditions. However, cell division or various external stressors can increase the permeability of the cell wall, allowing larger particles to enter. Although AgNPs larger than 20 nm in diameter can promote the formation of larger pores in the cell wall, allowing them to accumulate in the cell to a lesser extent [85,86], several studies have shown that significant size-dependent phytotoxic changes occur with NPs up to 20 nm in diameter [85,86,87,88,89,90,91]. Nevertheless, this altered permeability of the cell wall affects both the growth and morphology of algae, leading to pronounced negative effects [47,92]. Therefore, we cannot exclude that the initial size of the nanoparticles used had an influence on AgNP-induced phytotoxicity.

The transfer of AgNPs through aquatic ecosystems largely depends on the accumulation of AgNPs in algae [84]. In our study, all observed treatments resulted in internalization of silver into *C. vulgaris* itself to an extent that correlated with treatment concentrations, and the concentration of accumulated silver correlated well with the EC_25_ value obtained for each treatment.

Numerous studies on algae have investigated the effects of AgNPs on various parameters such as accumulation, uptake, and biotransformation of silver, as well as their effects on the growth and photosynthetic parameters [17]. However, there are only a limited number of studies that have investigated the occurrence of oxidative stress resulting from exposure of freshwater algae to AgNPs [41,42]. Monitoring the presence of oxidative stress is crucial because it plays an important role in the toxicity of AgNPs to biological organisms, mainly through ROS formation [11,17,75,93]. Therefore, understanding the mechanisms underlying oxidative stress in algae exposed to AgNPs is of great importance for assessing their potential ecological impact. In our study, all treatments resulted in a significant increase in ROS formation, which is consistent with previous studies with uncoated or citrate-coated AgNPs in *C. vulgaris* or *Chattonella marina* [40,94,95]. Considering that fluorescent probes DHE and H_2_DCFDA are indicators of formation of superoxide radical content and total ROS, respectively [57,96], our results show that negatively charged AgNP-citrate induce higher production of total ROS compared to AgNP-CTAB. Interestingly, treatments with AgNO_3_ showed very similar results to those with AgNP-citrate. On the other hand, the highest H_2_O_2_ production was measured upon exposure to AgNP-CTAB compared to AgNP-citrate, indicating a different phytotoxic mechanism of the differently stabilized AgNPs. For this parameter, exposure to AgNO_3_ resulted in values more similar to those of AgNP-CTAB, again indicating that the phytotoxicity of ionic silver is different from that of AgNPs. In addition, all treatments resulted in a significant increase in lipid peroxidation, which is consistent with studies conducted on *Chlorella pyrenoidosa* and *Poterioochromonas malhamensis* after AgNP or AgNO_3_ treatment [45,97], although AgNP-citrate and AgNO_3_ resulted in significantly higher levels compared to AgNP-CTAB. Moreover, protein carbonylation was also increased in treatments with AgNP-citrate and especially AgNO_3_ compared to AgNP-CTAB, which is another indication of their stronger phytotoxicity. Increased levels of protein carbonylation after exposure to AgNO_3_ are consistent with a previous study by Semerád et al. [98]. DNA damage, which to our knowledge was the first time studied in algal cells after exposure to AgNPs, was significantly induced by all treatments, although both types of AgNPs increased the percentage of tail DNA more than AgNO_3_, indicating a greater deleterious effect on DNA. Our results are consistent with the toxicity studies of other metal nanoparticles, in which treatment with iron, zinc, and copper oxides resulted in DNA damage in algae [99,100,101].

In response to the increased synthesis of ROS molecules due to exposure to AgNPs, numerous studies have shown that algae can activate their antioxidant system, which consists of enzymatic and non-enzymatic compounds, to mitigate the negative consequences of oxidative stress [42,43,45]. In our study, only AgNP-CTAB treatment resulted in a significant increase in the SOD activity, which correlates well with the results of DHE, which records the formation of superoxide radical and suggests that the increased activity of SOD effectively eliminates this toxic ROS compound by converting it to H_2_O_2_ [102]. Moreover, the increased activity of SOD only after treatment with AgNP-CTAB probably indicates a strong role of CTAB as a surfactant that inhibits possible interactions of AgNPs with other particles, as it has been shown that SOD and AgNPs can form complexes that alter the conformation of the enzyme and thus its activity [103]. Increased expression of SOD was also found in *C. reinhardtii* and *C. vulgaris* upon exposure to AgNPs [41,43]. The activities of PPX and APX were increased in all treatments, which is consistent with the results of previous studies [43,104]. However, the increase in PPX activity was more pronounced after exposure to AgNP-citrate and AgNO_3_ than to AgNP-CTAB. This result can be well correlated with H_2_O_2_ production, which was significantly increased in AgNP-CTAB treatment, in which PPX, which scavenges H_2_O_2_ and converts it to H_2_O, was not as efficient as in the other two treatments. These results once again demonstrate that the mechanism of toxicity of AgNP-citrate is more similar to that of AgNO_3_ than that of AgNP-CTAB, and that surface stabilizer plays an important role in the mechanism of toxicity. Only AgNO_3_ treatment induced an increase in the activity of CAT. The uninduced activity of catalase after treatments with both types of AgNPs in our study can be explained either by gene regulation [105] or, more likely, by a direct interaction of catalase and AgNPs which induced conformational changes in the CAT, thus resulting in an impairment of the CAT enzymatic activity [103].

The response to oxidative stress often involves the activation of non-enzymatic antioxidants such as proline and glutathione [75,106,107]. Proline accumulation was recorded after exposure of *Microcystis aeruginosa* to AgNP-PVP [108] and *P. malhamensis* to AgNP-citrate [97]. However, in our study, the proline content decreased after all treatments. These discrepancies between our results and those from the literature are probably due to different experimental conditions, i.e., Liu et al. [97] used much higher AgNP concentration and a shorter treatment time. The ratio of reduced (GSH) to oxidized (GSSG) glutathione is also an important indicator of oxidative stress attenuation, as GSH donates H^+^ ions and acts as a neutralizer of H_2_O_2_, which is subsequently oxidized to GSSG [109]. The GSH/GSSG ratio was significantly reduced to the same extent after all treatments, indicating that an attempt was made to reduce ROS-orchestrated stress conditions by H_2_O_2_ neutralization. The same results were obtained in *C. reinhardtii*, where the GSH/GSSG ratio significantly decreased after treatment, which is a significant indicator of AgNP-induced oxidative stress [72].

The final confirmation of the deleterious effects of all treatments is the stimulation of cell wall deformation and membrane destabilization, which was manifested by the appearance of plasmolysis. Treatments with both types of AgNPs caused increased plasmolysis compared with AgNO_3_ exposure, although AgNP-CTAB caused the greatest cell wall deformation. Zhao et al. [43] also showed that *C. reinhardtii* cells showed signs of plasmolysis after treatment with uncoated AgNPs, which could lead to membrane leakage and ultrastructural changes. Our results additionally confirm the above observations and are complemented by showing the influence of treatment with differently stabilized AgNPs, but also with AgNO_3_, on ultrastructural changes.

## 5. Conclusions

The effects of AgNPs on aquatic organisms are complex and depend on a variety of factors, both environmental and intrinsic. All treatments resulted in significant ROS formation that affected normal cell homeostasis, confirming that oxidative stress is the main mechanism of silver phytotoxicity, although stabilizers were shown to play an important role in AgNP phytotoxicity. However, the influence of the initial size of the AgNPs cannot be excluded. Analyses of AgNP stability in the exposure medium revealed higher agglomeration of AgNP-CTAB compared to AgNP-citrate, resulting in a higher concentration at which AgNP-CTAB allowed 75% of cells to survive. Furthermore, AgNP-CTAB showed less toxic effect on algal growth and caused weaker oxidative stress compared to AgNP-citrate, although it caused the most severe cell wall deformation. The AgNO_3_ stability test showed that released Ag^+^ ions reacted strongly with other ions in an ion-rich BBM medium, leading to the synthesis of newly formed AgNPs, and although AgNO_3_ was applied at the lowest silver concentration, it caused the fastest algal death. Interestingly, the extent of oxidative damage and antioxidant enzymatic defense when algal cells were treated with AgNO_3_ was similar to that when they were treated with AgNP-citrate. Moreover, the results showed that differently stabilized AgNPs caused different types of oxidative damage and activated different antioxidant mechanisms, as treatment with AgNP-CTAB resulted in increased H_2_O_2_ content, DNA damage, and SOD activity, whereas exposure to AgNP-citrate induced superoxide radical formation, lipid damage, and PPX activity. AgNO_3_ caused greater activation of the enzymatic antioxidants PPX and CAT, with a marked decrease in proline content. In summary, we have shown that differently stabilized AgNPs induce different effects in the algal cell, either by inducing different forms of ROS molecules or by different activity of the antioxidant response, suggesting that they result in different mechanisms of toxicity. Therefore, we provide clear evidence that AgNP stabilizers play a predominant role in AgNP-induced phytotoxicity to microalgae and highlight the importance of surface coatings in the analysis of AgNP phytotoxicity.

## Figures and Tables

**Table 1:** Silver content (µg 10^−6^ cells) in *C. vulgaris* cells after 72 h exposure to 0.188 mg L^−1^ AgNP citrate, 0.895 mg L^−1^ AgNP-CTAB, and 0.130 mg L^−1^ AgNO_3_ in a liquid BBM culture medium. The Ag content in the control cells was below the limit of quantification (<0.1 µg 10^−6^ cells). Values are given as the mean ± SE of three biological replicates. Treatments significantly different at *p* ≤ 0.05 (one-way ANOVA followed by a Newman–Keuls post hoc test) are indicated with different letters.

Treatment	µg 10^−6^ Cells
Control	0 ^d^
AgNP-citrate	15.62 ^a^
AgNP-CTAB	26.81 ^b^
AgNO_3_	9.96 ^c^

## Data Availability

The data used to support the findings of this study are included within the article.

## Supplementary Materials

The following supporting information can be downloaded at: https://www.mdpi.com/article/10.3390/nano13131967/s1, Figure S1: UV-Vis absorption spectra (A,E) and transmission electron micrographs of AgNP-citrate (B–D) and AgNP-CTAB (F–H) in stock solutions. Micrographs B and F—bright field image; C and G—silver element map; D and H—energy-dispersive X-ray spectrum. For each stock solution, four replicates (*n* = 4) were analysed; Figure S2: UV-Vis absorption spectra of 0.188 mg L^−1^ AgNP-citrate (A), 0.895 mg L^−1^ AgNP-CTAB (B), and 0.130 mg L^−1^ AgNO_3_ (C) after addition to a liquid BBM culture medium recorded over a period of three days; Table S1: Physicochemical properties of AgNP-citrate and AgNP-CTAB in stock solutions based on hydrodynamic diameter (*d*_H_) in nm determined from size distributions by volume, ζ-potential values in mV, and percentage of ionic Ag (Ag^+^); Table S2: Time evolution of changes in hydrodynamic diameter (*d*_H_) and zeta potential (ζ) of 0.188 mg L^−1^ AgNP-citrate, 0.895 mg L^−1^ AgNP-CTAB, and 0.130 mg L^−1^ AgNO_3_ after addition to a liquid BBM culture medium, recorded over a three-day period. Results are presented as volume size distributions and represent the mean ± SE of 10 measurements. The ζ-potentials are given as mean ± SE of 5 measurements.
