# Supplementary material for: The Occurrence of Oxidative Stress Induced by Silver Nanoparticles in Chlorella vulgaris Depends on the Surface-Stabilizing Agent
Source: Nanomaterials (Basel). 2023 Jun 28;13(13):1967. doi: 10.3390/nano13131967 (PMC10343332; doi:10.3390/nano13131967)
Supplement: Supplementary file 1 [file nanomaterials-13-01967-s001.zip › nanomaterials-2458870-supplementary.pdf]

nanomaterials

Supplementary Materials

The Occurrence of Oxidative Stress Induced by Silver Nanoparticles in *Chlorella vulgaris* Depends on the Surface-Stabilizing Agent

Bruno Komazec [1], Petra Cvjetko [1], Biljana Balen [1], Ilse Letofsky-Papst [2], Daniel Mark Lyons [3] and Petra Peharec Štefanić [1,*]

1 Department of Biology, Faculty of Science, University of Zagreb, Horvatovac 102a, 10000 Zagreb, Croatia; bruno.komazec@biol.pmf.unizg.hr (B.K.); pcvjetko@biol.pmf.unizg.hr (P.C.); bbalen@biol.pmf.unizg.hr (B.B.)
2 Institute of Electron Microscopy and Nanoanalysis (FELMI), Graz Centre for Electron Microscopy (ZFE), Austrian Cooperative Research (ACR), Graz University of Technology, Steyrergasse 17, 8010 Graz, Austria; ilse.papst@tugraz.at
3 Center for Marine Research, Ruđer Bošković Institute, G. Paliaga 5, 52210 Rovinj, Croatia; lyons@irb.hr
* Correspondence: ppeharec@biol.pmf.unizg.hr

Citation: Komazec, B.; Cvjetko, P.; Balen, B.; Letofsky-Papst, I.; Lyons, D.M.; Štefanić, P.P. The Occurrence of Oxidative Stress Induced by Silver Nanoparticles in *Chlorella vulgaris* Depends on the Surface-Stabilizing Agent. *Nanomaterials* **2023**, *13*, 1967. https://doi.org/10.3390/nano13131967

Academic Editors: Marta Marmiroli and Elena Maestri

Received: 2 June 2023
Revised: 23 June 2023
Accepted: 27 June 2023
Published: 28 June 2023

Copyright: © 2023 by the authors. Submitted for possible open access publication under the terms and conditions of the Creative Commons Attribution (CC BY) license (https://creativecommons.org/licenses/by/4.0/).

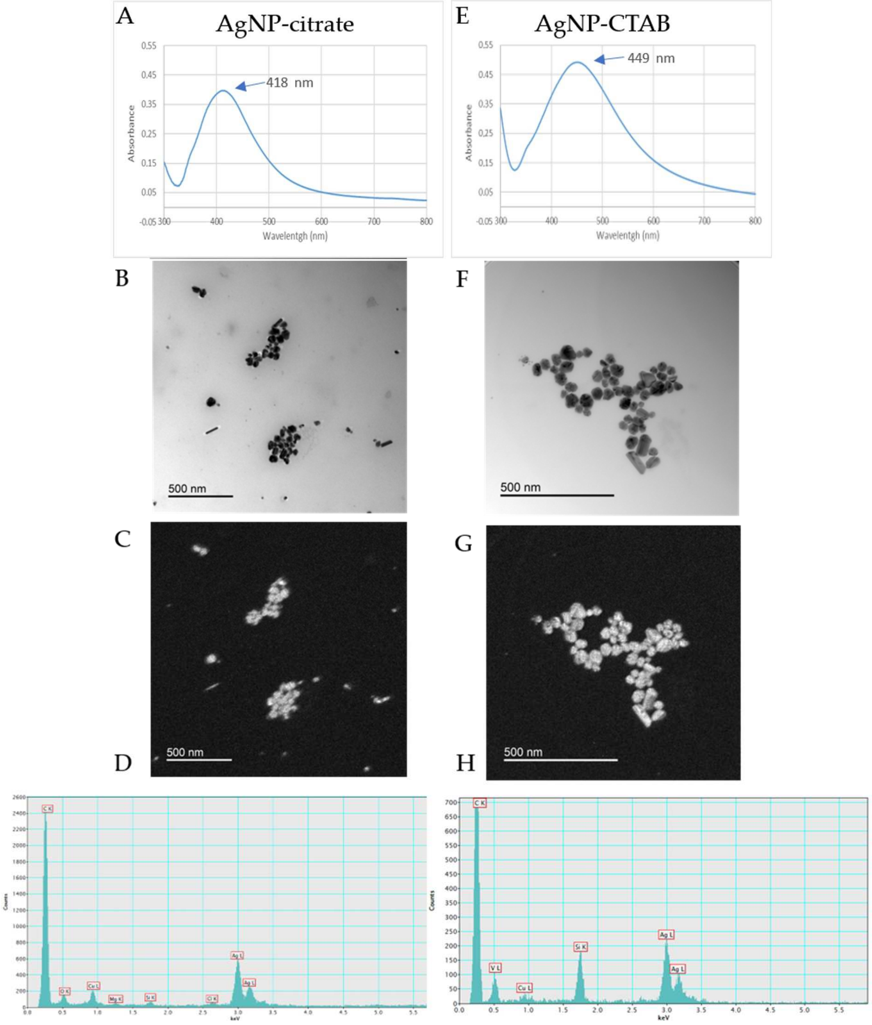

Figure S1. UV-Vis absorption spectra (**A**,**E**) and transmission electron micrographs of AgNP-citrate (**B**–**D**) and AgNP-CTAB (**F**–**H**) in stock solutions. Micrographs (**B**,**F**)—bright field image; (**C**,**G**)—

silver element map; (**D**,**H**)—energy-dispersive X-ray spectrum. For each stock solution, four replicates (n = 4) were analysed.

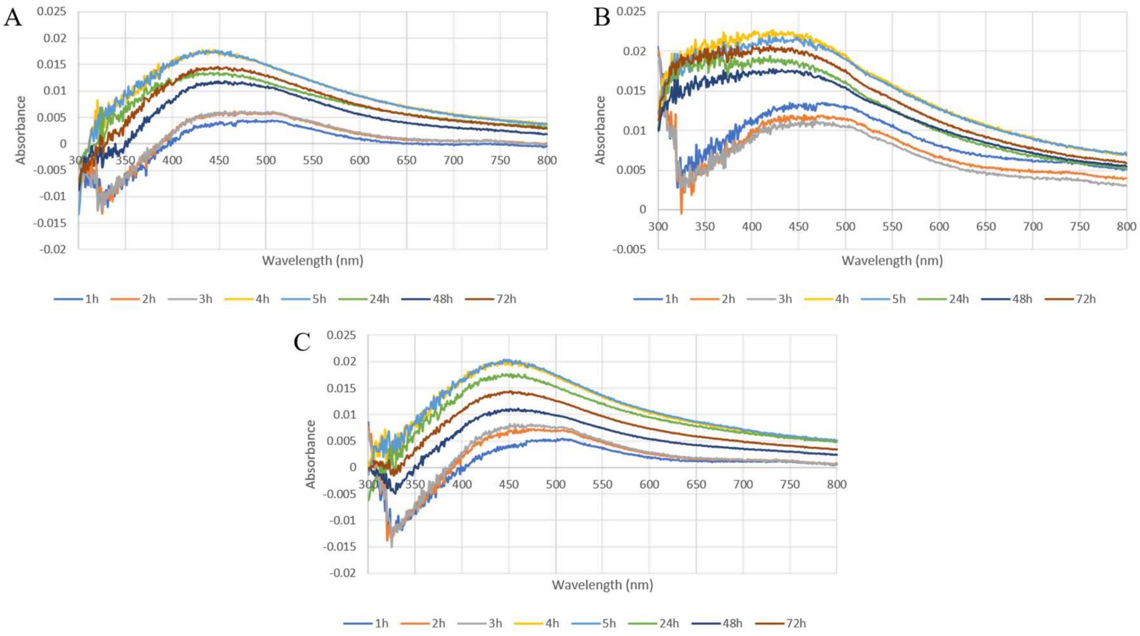

Figure S2. UV-Vis absorption spectra of 0.188 mg L^{-1} AgNP-citrate (**A**), 0.895 mg L^{-1} AgNP-CTAB (**B**), and 0.130 mg L^{-1} $AgNO_3$ (**C**) after addition to a liquid BBM culture medium recorded over a period of three days.

Table S1. Physicochemical properties of AgNP-citrate and AgNP-CTAB in stock solutions based on hydrodynamic diameter (d_H) in nm determined from size distributions by volume, ζ-potential values in mV, and percentage of ionic silver (Ag^+).

Treatment	AgNP-Citrate	AgNP-CTAB
Hydrodynamic diameter (d_H), nm	41.4 ± 0.9	82.8 ± 1.1
ζ potential, mV	−40.50 ± 3.21	51.34 ± 2.05
Concentration, mg L^{-1}	112.2	94.6
Ag^+, %	0.5	0.5

Table S2. Time evolution of changes in hydrodynamic diameter (d_H) and zeta potential (ζ) of 0.188 mg L^{-1} AgNP citrate, 0.895 mg L^{-1} AgNP-CTAB, and 0.130 mg L^{-1} $AgNO_3$ after addition to a liquid BBM culture medium, recorded over a three-day period. Results are presented as volume size distributions and represent the mean ± SE of 10 measurements. The ζ-potentials are given as mean ± SE of 5 measurements.

Time (h)	BBM Medium with AgNP-Citrate		BBM Medium with AgNP-CTAB		BBM Medium with $AgNO_3$	
	d_H (nm)	ζ Potential (mV)	d_H (nm)	ζ Potential (mV)	d_H (nm)	ζ Potential (mV)
0	109.6 ± 3.5	−29.23 ± 0.02	106.5 ± 1.1	−24.11 ± 3.70	174.7 ± 6.4	−11.17 ± 9.98
1	109.9 ± 2.6	−0.29 ± 0.06	115.6 ± 1.9	−23.14 ± 2.05	163.5 ± 5.3	−10.35 ± 14.56
2	114.9 ± 2.8	−35.89 ± 3.07	115.1 ± 1.3	−20.40 ± 1.87	161.3 ± 6.7	−27.46 ± 2.97
3	119.3 ± 1.5	−35.76 ± 4.20	114.7 ± 1.1	−25.13 ± 5.00	173.9 ± 3.7	−31.26 ± 2.74
4	113.4 ± 0.7	−29.43 ± 0.26	117.6 ± 2.4	−33.78 ± 4.25	161.9 ± 1.8	−15.62 ± 9.86
5	112.4 ± 2.5	−33.16 ± 7.18	118.0 ± 1.6	−24.93 ± 3.17	155.9 ± 4.4	−7.42 ± 12.88
24	68.0 ± 0.7	−25.97 ± 11.29	114.3 ± 1.7	−26.10 ± 2.20	94.9 ± 2.8	−26.76 ± 2.25
48	63.7 ± 0.9	−17.04 ± 9.32	114.3 ± 1.4	−26.92 ± 4.08	94.8 ± 4.1	−34.78 ± 3.58
72	61.77 ± 1.8	21.37 ± 5.42	131.5 ± 9.8	−10.01 ± 11.80	84.8 ± 8.2	0.00 ± 0.00
